# Transtubular Transoral Approach for Irreducible Ventral Craniovertebral Junction Compressive Pathologies: Surgical Technique and Outcome

**DOI:** 10.5704/MOJ.2307.006

**Published:** 2023-07

**Authors:** MH Ariffin, SN Mohd-Mahdi, A Baharudin, A M.Tamil, S Abdul-Rhani, K Ibrahim, BW Ng, JA Tan

**Affiliations:** 1 Department of Orthopaedics and Traumatology, Universiti Kebangsaan Malaysia, Kuala Lumpur, Malaysia; 2 Department of Anaesthesiology and Intensive Care, Universiti Kebangsaan Malaysia, Kuala Lumpur, Malaysia; 3 Department of Public Health, Universiti Kebangsaan Malaysia, Kuala Lumpur, Malaysia

**Keywords:** transoral approach, craniovertebral junction, myelopathy, os odontoideum, odontoidectomy

## Abstract

**Introduction:**

To investigate the use of a tubular retractor to provide access to the craniovertebral junction (CVJ) sparing the soft palate with the aim of reducing complications associated with traditional transoral approach but yet allowing adequate decompression of the CVJ.

**Materials and methods:**

Twelve consecutive patients with severe myelopathy (JOA-score less than 11) from ventral CVJ compression were operated between 2014-2020 using a tubular retractor assisted transoral decompression.

**Results:**

All patients improved neurologically statistically (p=0.02). There were no posterior pharynx wound infections or rhinolalia. There was one case with incomplete removal of the lateral wall of odontoid and one incidental durotomy.

**Conclusions:**

A Tubular retractor provides adequate access for decompression of the ventral compression of CVJ. As the tubular retractor pushed away the uvula, soft palate and pillars of the tonsils as it docked on the posterior pharyngeal wall, the traditional complications associated with traditional transoral procedures is completely avoided.

## Introduction

The transoral approach provides a direct route for removal of ventral compressive pathology of the craniovertebral junction (CVJ). The first series of irreducible atlantoaxial abnormality treated by transoral decompression was reported in 1962 by Fang and Ong^1^. However, the high morbidity and mortality caused many surgeons to shy away from the approach. The modern microsurgical approach to the CVJ must be credited to the work done by Menezes *et al*^2^, Spetzler *et al*^3^, and Crockard *et al*^4^. Menezes most importantly provided a detailed decision-making algorithm for the management of CVJ abnormality^5^. The key factors needed to be considered when treating the CVJ abnormality are (1) reducibility of the lesion (2) the site of encroachment into the CVJ (3) aetiology and (4) the growth potential of the lesion. In short, reducible lesion can be approached posteriorly while irreducible lesion with ventral compression must be approached ventrally. Despite the microsurgical advancements, the “access surgery” still carries significant “collateral damage” from splitting the soft palate such as rhinolalia, nasal regurgitation and palatal wound dehiscence. Jones *et al* reported oropharyngeal complications in 75% patients who had undergone splitting of soft palate compared to 15.4% of patients who did not undergo splitting of the soft palate^6^. Ariffin *et al* reported the use of a tubular retractor to provide an “access surgery” for microsurgical transoral excision of os odontoideum by inserting the right length tubular retractor directly into the mouth obviating the need to split the soft palate as the tubular retractor naturally pushes away the soft palate, the uvula and the pillars of the tonsils when its “docks” down on the oropharynx^7^. The surgery was complicated by durotomy managed by tissue sealant. No approach- related complications occurred. Clinically the patient improved neurologically with post-op CT scan showing adequate decompression. Similar use of a tubular retractor has also been reported by Zaninovich *et al* in transoral odontoidectomy for abscess drainage and phlegmon resection in a patient with progressive cervical myelopathy. Their patient had neurological improvement only in the upper limbs. There are no access related complications. They concluded that it may serve for access to other pathologies in this location as well^8^.

In this report we present our experience with tubular retractor assisted transoral surgery and its refinements with the introduction of newer surgical adjuncts (navigation and endoscope) aimed at ensuring safer surgery and complete resection of the ventral compression on the CVJ. Our aim was to assess the results (adequate decompression assessed by intra operative CT scan / post-operative CT scan, postoperative clinical examination of the neurological deficit (JOA score), difficulties encountered and complications of using a tubular retractor assisted transoral approach. We postulated that tubular retractor- assisted transoral approach will reduce the specific complications associated with the traditional transoral approach and allow adequate decompression with subsequent improvement in neurological function.

## Materials and Methods

The indication for tubular retractor assisted transoral surgery was irreducible ventral compression of the CVJ with myelopathy. It is contraindicated when the compression extended inferiorly beyond the C2,3 disc; the distance between the upper incisors to the posterior pharyngeal wall is more than 100mm on the MRI (maximum tube length 100mm) and an inability to open the mouth more than 2.5cm (tube diameter 26mm). Between 2014-2020, 12 consecutive patients presenting with myelopathy from ventral CVJ compression were operated. The first six patients were operated using the tubular retractor [METRx, Medtronic Sofamor Danek, Memphis, TN] as an access, and microsurgical technique [Pentero 900, Carl Zeiss Meditec, Germany]. The last six patients were operated using tubular retractor with exoscope with 3D4K digital hybrid visualisation [Kinevo 900, Carl Zeiss Meditec, Germany], O-arm navigation [StealthStation, Medtronic, Minneapolis, MN, USA] and endoscope [Qevo, Carl Zeiss Meditec, Germany].

There were eight males and four females. Although the aetiology for the compressive pathology varied, a common feature on MRI findings in all the patients were severe ventrally directed spinal cord compression with marked limitation of the space available for the cord. The patient demographic, diagnosis, and JOA score were presented in (Table I).

**Table I:** Demographic data of patients operated using tubular transoral approach

Patient No.	Age	Gender	Compressive Pathology	Treatment	JOA pre-op	JOA post-op
1	15	Male	Os odontoideum	Odontoidectomy with C1C2 fusion	6	15
2	54	Female	Rheumatoid arthritis	Odontoidectomy with OC fusion	8	15
3	45	Female	Rheumatoid arthritis	Odontoidectomy and OC fusion	7	13
4	52	Female	Rheumatoid Arthritis	Odontoidectomy with OC fusion	7	11
5	40	Male	Hypertrophic Odontoid Non-union	Odontoidectomy with C1C2 fusion	6	13
6	62	Female	Rheumatoid Arthritis	Odontoidectomy with OC fusion	7	16
7	16	Male	Os odontoideum	Odontoidectomy with C1C2 fusion	6	15
8	15	Male	Larsen Syndrome C1C2 type 3 Rotatory Dislocation with severe kyphoscoliosis	Odontoidectomy with C1C2 fusion with scoliosis surgery 3 months later	6	15
9	22	Male	Os odontoideum	Odontoidectomy with C1C2 fusion	7	15
10	12	Female	Goldenhar Syndrome with basilar invagination	Odontoidectomy with C1C2 fusion	7	15
11	60	Male	Hypertrophic odontoid Non-union	Odontoidectomy with C1C2 fusion	6	15
12	16	Male	C2 Aneurysmal bone cyst	Odontoidectomy (Previous OC fusion)	8	15

All patients were intubated by an awake fibreoptic technique, and a throat pack inserted. Blood pressure was maintained within normal limits to maintain cord perfusion in the presence of severe myelopathy. Tranexamic acid (15-20mg/kg body weight) was given. A Ryle’s tube was inserted for post-operative feeding. Antibiotic prophylaxis given was a combination of Ceftriaxone and Metronidazole which were continued till day 5 post-op. Intra operative neuromonitoring was used in all cases.

For illustration, we provide the surgical procedure for a 16-year-old boy who was diagnosed with Larsen Syndrome and was referred to us for severe kyphoscoliosis with progressive weakness of right upper limb. He was unable to grip his cup, unable to button and unbutton his shirt and has limited range of motion of the neck. He was able to walk and had no urinary and bowel incontinence. The patient had delay of gross motor skills during childhood (crawled at the age of two and walked at four)

**Fig 1:**
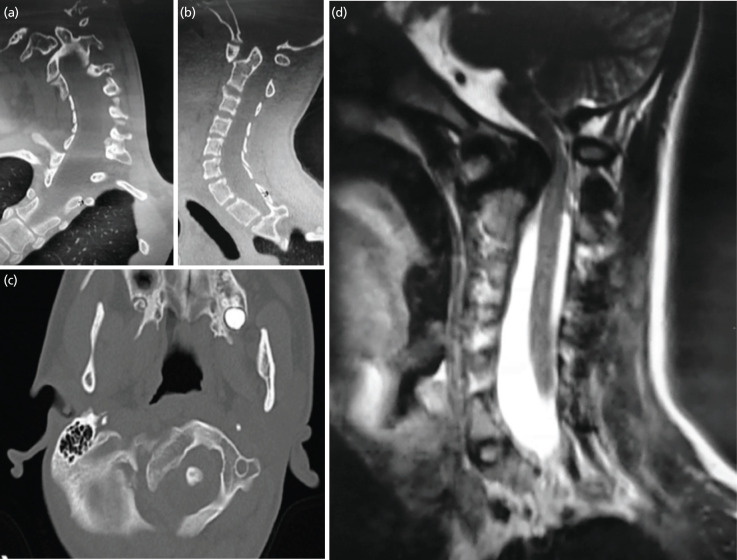
CT of the cervical spine with coronal (a), sagittal (b) and axial (c) reconstruction and MRI of the cervical spine in sagittal T2-weighted (d). CT demonstrates narrowing of the spinal canal of 50% with atlantoaxial dense interval (ADI) of 9mm, borderline basilar invagination where the tip of the odontoid process just touches the McRae line and type III atlantoaxial dislocation. MRI reveals an intramedullary high signal intensity at the region of compression indicating myelomalacia. The spinal canal is severely narrowed that the normal CSF anterior and posterior to the spinal cord is compromised.

Clinically, he had severe thoracic kyphoscoliosis with a huge right rib hump, dysmorphic neck features and hyperlaxity of joints. He walked unaided with a broad-based gait. Neurologically his right upper limb was weak with motor power of C5: 3/5, C6: 3/5, C7: 2/5, C8: 3/5, and T1: 3/5. The reflexes were brisk. All dermatomal sensations were intact. Patient subsequently underwent CT and MRI investigations for pre-operative planning (Fig. 1).

**Fig 2:**
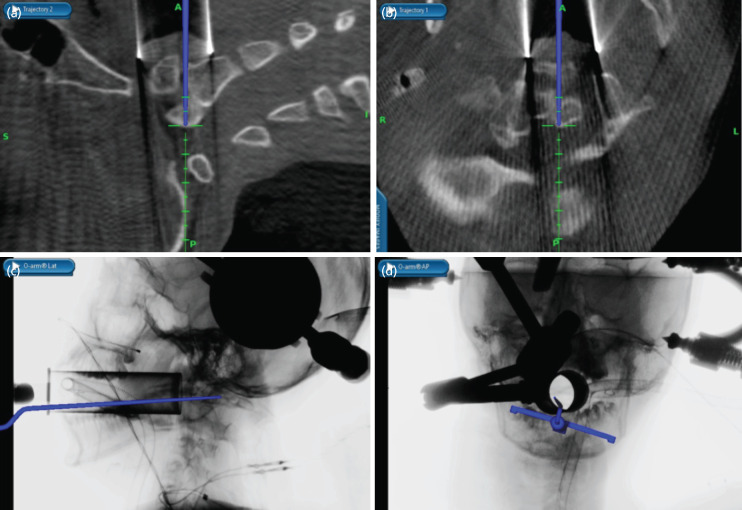
Intra-operative navigation used to identify the tip of odontoid. (a, b) shows the identification of odontoid with the use of passive probe in the sagittal and axial views, respectively. (c, d) Shows the position of the tube and passive probe in AP and lateral radiograph taken by the O-arm.

The patient underwent tubular retractor assisted navigated transoral odontoidectomy with C1C2 dorsal wiring. Awake fibreoptic intubation was performed and a flexometallic endotracheal tube was secured. A Ryle’s tube was inserted for future feeding. The head was secured to a Mayfield clamp. The articulating arm for the cranial reference frame was then attached to the Mayfield. He was positioned supine with a 30° head-up inclination. After cleaning the mouth with povidone iodine, the area was draped exposing only the mouth. The cranial reference frame was attached to the articulating arm ensuring that it was captured by the sensors. The second dilator of the METRx system was used to determine the length of the tube and then a 90-mm tubular retractor with diameter of 26mm was inserted through the oral cavity under direct visualisation ensuring the tube was pushed up past the soft palate and uvula before docking on the posterior pharyngeal wall. An AP and lateral fluoroscopic images were then obtained with the O-arm to confirm tube placement on C1 anterior arch. Because of the AARD and a scoliotic cervical spine, the exact position of the tip of odontoid was difficult to determine. To identify the exact location, an intraoperative CT scan was performed with O-arm and the images were used for navigation. A passive planar blunt probe was used to point to the exact location of the dislocated odontoid tip and the METRx tube was adjusted to centre on the probe (Fig. 2).

The tube, docked down on the pharyngeal wall exactly centered on the dislocated odontoid was secured to the side rail of the operating table. An operating exoscope with 3D4K hybrid visualisation [Kinevo 900, Carl Zeiss Meditec, Germany] provides visualisation, magnification, illumination and excellent depth perception.

**Fig 3:**
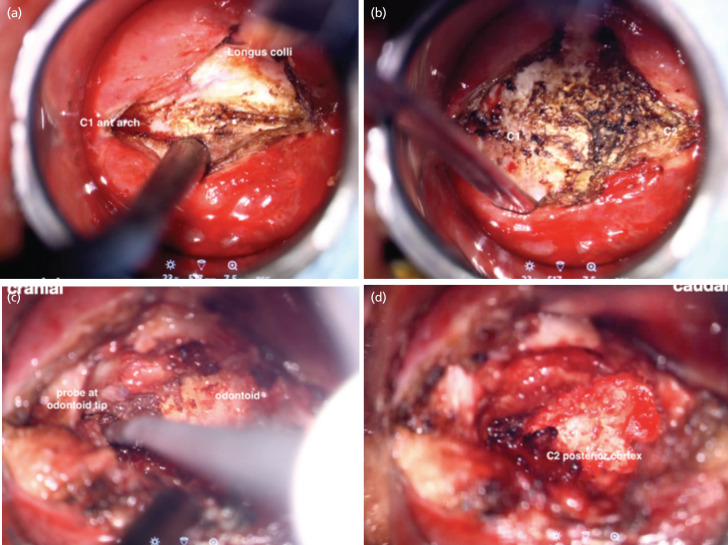
(a) Key surgical landmarks after the incision of the posterior pharyngeal wall includes tubercle on C1 anterior arch for midline identification, (b) exposure of the C1 arch, (c) identification of odontoid tip using the passive probe after the removal of C1 arch and (d) visualisation of C2 posterior cortex after the removal of anterior odontoid bone with high-speed burr.

**Fig 4:**
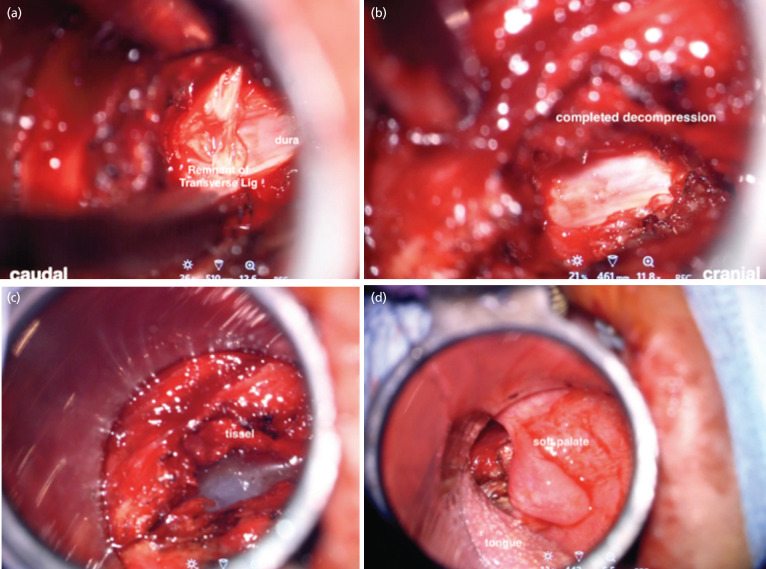
(a) Remnant of transverse ligament can be seen after the removal posterior wall of odontoid using a Kerrison rongeur. (b) Completed decompression. (c) Tissel was used for closure during removal of the tube the tongue, (d) soft palate and uvula was visually inspected ensuring no injury.

A midline incision was made over the posterior pharyngeal wall to expose the anterior tubercle of the C1. To access the dens, the middle portion of the anterior arch of C1 was removed with a high-speed burr. From time-to-time navigation was performed using the passive planar blunt probe to accurately locate the pathoanatomy, ensuring a safe and direct access to the odontoid peg. The surgeon needed to change position from left to the right side to ensure complete removal of the lateral wall of the odontoid during the surgery. The posterior wall of the odontoid was removed with Kerrison, and the spinal cord decompression was completed with the removal of the transverse ligament and parts of the alar ligament. At this stage an endoscope was utilised to pick up any residual compression not seen on the exoscopic view. A final intra-operative CT scan was performed ensuring a complete decompression. The 2cm posterior pharyngeal opening was closed with Tisseel [Baxter Healthcare Corp, Glendale, CA]. As the METRx tube was removed visual inspection was done ensuring no damage to the uvula, soft palate and the tongue. The key surgical landmarks identified microsurgically were depicted in (Fig. 3 and Fig. 4). The blood loss was 270ml.

A hard collar was applied, and the patient was then placed in a prone position for C1-C2 dorsal wiring technique. The whole procedure lasted 220 minutes. The patient was sent to the Post Anaesthesia Care Unit post-op and extubated the next day. He was started on Ryle’s tube feeding on the second post-operative day. The oral hygiene was maintained with two-hourly Thymol gargles. The patient regained full power of his right upper limb with the exception of T1 root, with no sensory deficit bilaterally in the upper limbs. He was able to ambulate with a forearm support by post-op day 4. He was able to drink with his right hand holding a cup on post-op day 5. Direct laryngoscopy performed post-operatively on day 3 showed minimal slough and on day 5 revealed a clean granulating wound without infection. Cold liquid diet was started on post-op day 5. Patient went home on day 6 post-op. At two weeks review in outpatient clinic, the anterior and posterior wound had healed, and patient was allow oral solid food intake.

The patient successfully underwent posterior scoliosis surgery three months after the transoral decompression. During follow-up after six months, his condition had improved with an ability to feed and button-unbutton his shirt himself. He is now two years post scoliosis surgery.

## Results

All patients except case no. 9 were extubated the next day and sent to the ward. Average length of stay post-operatively was six days (range 5-10 days). All patients showed statistically significant neurological improvement. The data were analysed using Wilcoxon Signed Rank test and yielded a p-value of 0.002. No post-operative posterior pharyngeal wound infection occured. No dysphagia, dysphonia, and nasal regurgitation of fluids was noted. One incidental durotomy occurred and was managed by tissue sealant to stop the CSF leak. Post-operative CT scan in the first six patients showed adequate decompression except in case no. 4 where the lateral wall of the odontoid was incompletely removed. Combination of the endoscope and intraoperative CT scans in the remaining six patients ensured no residual compression of the CVJ. We did not do a post-operative MRI. In all cases a good bone fusion was accomplished as demonstrated by post-operative radiographs.

The difficulty that we encountered were (1) adapting to use the instruments via a long working tube making sure the hands and the instruments do not block the target surgery area. This is fundamental when working with a long tubular retractor and was overcome with practice. A few tricks like bending 90° the long diathermy, use of angled/bayonet instruments and curved or angled burr helped. (2) Incomplete removal of the lateral wall of odontoid (case no. 4) on post-op CT scan. When the surgeon stood on the right, he overlooked that the right lateral wall was not completely removed on what seemed to be adequate decompression with the microscope. This was solved subsequently by working on both sides of the patient ensuring both lateral wall of odontoid was completely visualised and removed. The introduction of the endoscope subsequently also solved the problem of incomplete bony removal as with the 45° camera it allowed us to visualise all around. In case no. 9 in the presence of basilar invagination the endoscope clearly picked up the incompletely removed tip of odontoid. Additional intra op CT also eliminated the problem of inadequate bony decompression. (3) Suturing the posterior pharyngeal wall. Suturing the posterior pharyngeal wall was assisted by using an arthroscopic grasper as needle holder and a knot pusher to facilitate securing knots via a long tube. In case no. 7,8 and 9 we used Tisseel [Baxter Healthcare Corp, Glendale, CA] to cover the wound. In these cases, the opening on the posterior pharyngeal wall was less than 2cm as navigation was used to precisely locate the bony compression and hence smaller opening on the posterior pharynx.

## Discussion

The introduction of tubular retractor with angled instruments have revolutionised minimally invasive lumbar spine surgery (MISS). One of the key principles of MISS is minimal “access surgery” related collateral damage yet maintaining the same treatment of the “target surgery”. From our experience with tubular retractor in MISS we adapted its usage to provide an access which is a simple, fast and safe technique as the approach is made by inserting the right length tubular retractor direct into the mouth pushing up the uvula and soft palate as it docked on the posterior pharyngeal wall hence minimising the typical morbidity to the soft palate associated with formal transoral approach. Additionally, the tube also protects the tongue, soft and hard palate, tonsillar areas that can be injured by the instruments during traditional transoral as the tube acted as a working portal. Pre-operative factors taken into consideration when attempting to use tubular retractors were (1) the width of mouth opening between the two incisors should be at-least 2.5cm to accommodate the size of the tube used. Technically using a smaller tube is feasible but working with the instruments via a long working tube is technically more demanding. (2) The length of the tube would be measured pre-operatively by measuring on the MRI the distance between the incisors and the posterior pharyngeal wall. The length of the tubes used were between 80mm to 100mm. In an anatomic study evaluating the anterior of C1-C2, Tun *et al* have reported that the widest odontoid diameter on the coronal plane was 10.1 +/- 1.4mm with a minimum drilling diameter of 10.8 +/- 1.1mm of the anterior ring removal^9^. We believe measuring this is important at pre-operative planning as it will ensure that we will be comfortable working on the lesion through the tube. If the lesion is bigger than the tubular access, more “wanding” will be required further increasing angulation of the tube, the exoscope and technical difficulty.

Another new concept in visualisation introduced in this study was using an exoscope with 3D4K hybrid visualisation. As the tube is typically docked at an angle to access the pathology of the CVJ using the exoscope allowed the surgeons and assistants to be independent and free of the oculars and allowed them to operate in more ergonomic position as visualisations were provided by 3D4K monitors^10^.

The use of an endoscope for transoral surgery has been well documented and is an established approach^11,12^. The endoscope used in this study was a 45° endoscope [Qevo, Carl Zeiss]. It is designed to be plugged in and directly integrates into KINEVO 900 digital visualisation platform. It allowed us to “look around the corners” which is not seen on the microscope/exoscope platform as an endoscope has a bigger surgical exposure volume compared to a surgical microscope^13^. Additionally, it allowed a streamlined approach in the operating theatre by eliminating the need of a dedicated endoscopic tower. In a cadaveric study, Karl-Michael *et al* concluded that this innovative, handheld, endoscopic tool allows excellent additional visualisation of the surgical field and effectively enhances the modern neurosurgical armamentarium^14^.

The application of navigation has been useful in both cranial and spinal surgeries because it provides three-dimensional spatial orientation during surgery^14,15^. When dealing with basilar invagination and atlantoaxial dislocation with abnormal anatomy, navigation affords higher precision and accuracy to help with the odontoidectomy^16^. Navigation also decreases the need for intra-operative fluoroscopy, hence decreases the amount of radiation exposure both to the patient and to the operating room staffs.

## Conclusions

In summary, this report describes the indication, surgical technique, result, difficulties and complications associated with use of tubular retractor assisted transoral decompression. In irreducible ventral compressive pathology of the CVJ, the transoral route provides the most direct access for effective decompression. For a select lesion confined to the C2 and odontoid, tubular assisted surgery is safe as it minimises the access related complications typically associated with splitting of soft palate in traditional transoral approach. On top of complication reduction and avoidance, the tubular retractor augmented with exoscope and surgical navigation provides a minimally invasive access, minimal collateral damage, better surgical precision and decompression, surgeon ergonomics, patient safety with excellent post-operative outcome. We hope this report will spark an interest in using a tubular retractor as a minimally invasive approach for selected ventral CVJ compressive pathology.
